# Sol-Gel Films Doped with Enzymes and Banana Crude Extract as Sensing Materials for Spectrophotometric Determination

**DOI:** 10.3390/gels9030240

**Published:** 2023-03-18

**Authors:** Maria A. Morosanova, Elena I. Morosanova

**Affiliations:** Analytical Chemistry Division, Chemistry Department, Lomonosov Moscow State University, 119234 Moscow, Russia

**Keywords:** sol-gel films, optical sensors, tetraethoxysilane, phenyltriethoxysilane, silicon polyethylene glycol, peroxidase, tyrosinase, crude banana extract, determination of total polyphenol content

## Abstract

Chromogenic enzymatic reactions are very convenient for the determination of various biochemically active compounds. Sol-gel films are a promising platform for biosensor development. The creation of sol-gel films with immobilized enzymes deserves attention as an effective way to create optical biosensors. In the present work, the conditions are selected to obtain sol-gel films doped with horseradish peroxidase (HRP), mushroom tyrosinase (MT) and crude banana extract (BE), inside the polystyrene spectrophotometric cuvettes. Two procedures are proposed: the use of tetraethoxysilane-phenyltriethoxysilane (TEOS-PhTEOS) mixture as precursor, as well as the use of silicon polyethylene glycol (SPG).In both types of films, the enzymatic activity of HRP, MT, and BE is preserved. Based on the kinetics study of enzymatic reactions catalyzed by sol-gel films doped with HRP, MT, and BE, we found that encapsulation in the TEOS-PhTEOS films affects the enzymatic activity to a lesser extent compared to encapsulation in SPG films. Immobilization affects BE significantly less than MT and HRP. The Michaelis constant for BE encapsulated in TEOS-PhTEOS films almost does not differ from the Michaelis constant for a non-immobilized BE. The proposed sol-gel films allow determining hydrogen peroxide in the range of 0.2–3.5 mM (HRP containing film in the presence of TMB), and caffeic acid in the ranges of 0.5–10.0 mM and 2.0–10.0 mM (MT- and BE-containing films, respectively). BE-containing films have been used to determine the total polyphenol content of coffee in caffeic acid equivalents; the results of the analysis are in good agreement with the results obtained using an independent method of determination. These films are highly stable and can be stored without the loss of activity for 2 months at +4 °C and 2 weeks at +25 °C.

## 1. Introduction

Sol-gel materials are widely used in analytical practice. The immobilization of proteins in sol-gel matrices has been attempted in a number of works, given the good properties of the resulting materials: preservation of the biocatalytic properties of the encapsulated proteins and excellent optical properties. Since the first reported case of a successful immobilization of active alkaline phosphatase via the sol-gel method [1], sol-gel silicate has become a desired immobilization matrix for the design of active biocomposite materials. Sol-gel materials as a matrix for immobilized proteins or biomolecules can have many applications, such as stationary phase [2], drug delivery materials [3], and coatings [4,5]. Often sol-gel materials with immobilized enzymes are created for analytical enzymatic applications [1,6,7,8,9,10,11,12,13,14,15].

Silica sol-gel films present a major advantage comparing to other sol-gel materials for enzyme immobilization: the closeness of the immobilized enzymes to the solid-solution interface. This increases the accessibility of the enzymes to substrates from the aqueous phase, making silica sol-gel film a promising platform for biosensor development. Sol-gel films are particularly often employed for both optical [6,7,8,9,10,11,12], and electrochemical biosensors [6,13,14,15].

Chromogenic enzymatic reactions are very convenient for the determination of various biochemically active compounds. In our opinion, the use of sol-gel films to create optical biosensors deserves attention as an effective way to simplify analysis for its further implementation in field conditions.

To obtain sol-gel materials doped with biomolecules, the latter are often encapsulated in matrices of Ormosils—materials obtained by the hydrolysis of tetraethoxysilane in the presence of organic silicon alkoxides, primarily 3-aminopropyltriethoxysilane [16,17]. However, the alcohol formed during the hydrolysis and condensation of the alkoxide precursors could negatively affect the entrapped enzymes’ activity [17]. This is an important problem that must be solved before employing the sol-gel process as a universal method of protein or other biomolecules encapsulation. In the literature, special schemes are described for the synthesis of sol-gel materials doped with enzymes to preserve the enzymatic activity of immobilized enzymes [18,19,20,21,22,23]. The approaches described can be divided into two groups: the use of tetraethoxysilane and its derivatives as precursors while using various ways to minimize the contact of the enzyme with ethanol released upon hydrolysis of the precursors and the use of silicate and glycerates as precursors, the hydrolysis of which does not release ethanol.

The approaches in the first group include the modification of the procedure while using standard alkoxide precursors [9,10,18]. To minimize the contact of the enzyme with ethanol, a two-stage synthesis scheme is proposed: the first stage is the hydrolysis of tetraethoxysilane or its mixtures with organic silicon alkoxides and the preparation of the sol; the second stage is the addition of the enzyme to the sol-gel solution and the formation of gels. Sometimes even the removal of the alcohol by rotavaporization method is performed before the second stage [18]. The technology of “kinetic doping” is also proposed where the nascent sol-gel film is submerged into enzyme-containing buffer solution which provides alcohol dilution [9,10].

The second group implies the use of different precursors: sodium silicate [19,20] or glycerol-derived silicates [21,22,23], which provide an alcohol-free sol. Both these routes possess some limitations in their application: the glycerol-derived silicate precursors need to be synthesized and sodium silicate precursors produce high sodium concentration levels in the sol.

Using crude extracts as enzyme sources in biocomposite sol-gel materials seems a promising approach. It was shown for crude extracts with polyphenol oxidase activity that, other conditions being equal, the enzymatic activity of sol-gel materials doped with the extracts is significantly higher than the activity of sol-gel materials doped with commercial tyrosinase [11,12]. Based on our experience of studying crude extracts, we also noted that the use of plant and mushroom extracts as enzyme sources presents a number of advantages compared to purified enzymes: higher interference thresholds, better stability, and lower cost [24,25,26]. In our opinion, the study of the crude extracts’ properties and the study of the possibilities of their inclusion in sol-gel films will contribute to the development of methods for the field determination of biochemically important analytes.

Historically, sol-gel films for optical applications were prepared on glass slides, which then could be put inside the cuvettes. However, a more efficient and practical way to create an optical biocomposite sensor is also described [27,28]: the sol-enzyme mixture is put directly into the dispensable polystyrene cuvettes and the film is formed on the cuvette inner side. This approach provides an easy way to fixate the film position in relation to the optical path and also to precisely measure the amount of the enzyme and sol.

The goal of this work was to develop methods for the synthesis of transparent sol-gel films doped with the most well-studied enzymes (horseradish peroxidase and mushroom tyrosinase), as well as with crude banana extract as a source of polyphenol oxidase, on the inner surface of plastic cuvettes with the use of tetraethoxysilane and organic alkoxides or silicon glycerate as precursors, studying the effect of immobilization on the activity of these enzymes, and evaluating the analytical performance of synthesized sol-gel films.

## 2. Results

### 2.1. Synthesis of Sol-Gel Films with Immobilized Enzymes and Banana Extract

The validity of the sol-gel route presented in this work to preserve the enzyme activity during the immobilization process has been studied on two of the enzymes that are most widely used in analytical methods: horseradish peroxidase (HRP) and mushroom tyrosinase (MT), and also the widely used crude plant extract—banana extract (BE) as a source of polyphenol oxidase [23]. Most sol-gel immobilization studies use HRP [9,10,15,18,19,21]; polyphenol oxidase (tyrosinase) is studied less frequently [11,12,13,14,29]. We have optimized the sol-gel matrix using HRP, and then studied the performance of HRP, MT, and BE in the selected conditions. We have developed two approaches: using sol-gel films based on mixtures of TEOS with derivatives (i.e., Ormosils) and using glycerol precursors; then we studied the effect of immobilization on the activity of enzymes and the possibility of analytical use of the synthesized films.

#### 2.1.1. Sol-Gel Films Based on Alkoxide Precursors (TEOS Films)

Sol-gel synthesis consists of successively carrying out the following stages: hydrolysis of precursors, polymerization (transformation of a sol into a gel) and, if necessary, drying of the gels under different conditions. Typically, hydrolysis is carried out in the presence of a related alcohol; when using TEOS, in the presence of ethanol. The properties of sol-gel materials depend on the nature of the precursors, the ratio of components in the hydrolyzing mixture, the nature of the gelation catalyst, and special additives to control the porosity of the materials. To obtain sol-gel materials doped with analytical reagents, we have developed a synthesis scheme [30,31], based on the hydrolysis of tetraethoxysilane in an aqueous-ethanol medium in the presence of hydrochloric acid as a catalyst and cetylpyridinium chloride as a pore former [32]. This scheme was used as the basis for the development of a method for the synthesis of sol-gel films doped with enzymes.

Studies show that when enzymes are included in Ormosils, i.e., sol-gel materials obtained from modified alkoxide precursors, their activity decreases to a lesser extent than in the case of standard TEOS materials. Most often, methyl- and amino-derivatives of tetraethoxysilane are used as precursors for the immobilization of enzymes in sol–gel materials [16], while phenyl derivatives are used much less frequently.

According to the literature, adding the enzyme solution to the already formed sol and not to the hydrolyzing mixture seems like a promising approach. This method was proposed using sodium silicate as a precursor [19]. We decided to take this approach when we used tetraethoxysylane (TEOS) and its mixtures with phenyltriethoxysilane (PhTEOS) and 3-aminopropyltriethoxysilane (AmTEOS) as sol-gel precursors. These TEOS-based sol-gel matrices were studied using horseradish peroxidase (HRP) as a model enzyme. HRP is widely used in enzyme immobilization studies and comparing the results of the present study using HRP was likely to be easier.

In order to select the conditions for the synthesis of transparent HRP-containing sol-gel films on the inner surface of plastic cuvettes, the influence of the nature and ratio of precursors, concentrations of hydrochloric acid and cetylpyridinium chloride was studied. TEOS, AmTEOS, and PhTEOS were used as precursors. The mixtures of precursors with water were prepared with 3:1 ratio of precursors:water and were mixed under the influence of ultrasound with a sound energy density of 0.24–0.38 W/mL at the hydrolyzing stage. The transparent homogeneous sols were formed in 45–90 min. The resulting sols are stable and can be stored at +4 °C for a week. To obtain gels, the sols were mixed with HRP solution in a buffer (pH 6.0) in a ratio of 1:0.8. The loss of fluidity of this mixture (i.e., gel formation) occurs after 2–10 min, depending on the composition of the sol. To prepare the sol-gel films, 0.5–0.9 mL of the mixture was placed in cuvettes, distributed on one of the inner sides, and after 2–10 min, films with an approximate thickness of 1.5–2.5 mm were formed. The stability of the sol-gel films, the enzymatic activity of the immobilized enzymes, and their storage stability were evaluated to choose the synthesis conditions.

When 1.0–4.5% *w*/*w* AmTEOS was added to TEOS, the films lost transparency compared to films prepared with only TEOS. For TEOS-AmTEOS films, the 10–100 fold increase in the concentration of hydrochloric acid led to the increase in transparency, but it was accompanied by significant reduction in the gelation time (less than 1 min), which made it difficult to obtain films by our method.

The use of TEOS-PhTEOS mixtures with a PhTEOS content of 1–10% *w/w* made it possible to obtain films that are transparent in the visible light range. Films produced at PhTEOS contents greater than 2% cracked on the next day after the preparation, but at 1–2% *w*/*w* they remained stable and did not crack. When using mixtures of TEOS-AmTEOS-PhTEOS with 1% *w*/*w* of PhTEOS and 0.4% *w*/*w* of AmTEOS, a lack of film transparency was observed, which led us to stop using AmTEOS and to concentrate on studying TEOS-PhTEOS mixtures.

**Figure 1:**
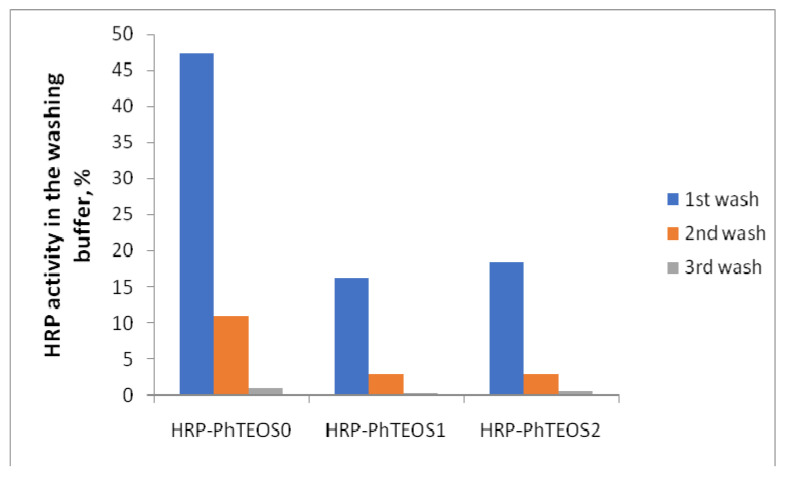
HRP activity (% in relation to the initial TEOS-PhTEOS sol-gel film activity) in the washing buffer solutions (pH 6.0).

In the literature, enzyme-doped sol-gel materials obtained by various methods are usually characterized by the retention of the immobilized enzyme in the matrix and its activity [9,19,21]. We investigated the effect of PhTEOS content on enzyme retention using HRP as a model enzyme. Under the described above conditions, HRP-doped films were synthesized with different PhTEOS content in the mixture of precursors (0, 1, and 2%)—HRP-PhTEOS0, HRP-PhTEOS1, and HRP-PhTEOS2 films. Enzyme retention was studied by determining the enzyme activity in buffer solutions obtained by washing the sol-gel films. The results are shown in Figure 1. The introduction of PhTEOS into the precursor mixture increased the retention of peroxidase by the sol-gel matrix. Thus, it can be concluded that peroxidase retention is significantly improved when PhTEOS is introduced into the matrix, and the enzyme is washed out slightly less from films containing 1% PhTEOS than from the films containing 2% PhTEOS. The obtained HRP retention values in the films after three washes are shown in Table 1. Comparison with literature data shows that the enzyme washing out values in our experiments are somewhat greater than for the previously proposed methods [19,21]. However, our film preparation method is simple, involves the use of commercially available precursors, and preserves significant activity of the immobilized enzyme.

It is widely known that the activity of an entrapped enzyme is usually only a fraction of its activity in free solution [9]. The relative activity of the immobilized enzyme was calculated as a percentage of the activity of a similar amount of the enzyme in solution. The initial rates comparison allowed calculating the film loaded enzymes’ relative activity (Table 2). The relative activities were in the range of 6.6–7.4%, which is similar to the results observed for other sol-gel matrices described in the literature [9,19]. The highest relative activity was observed for 1% PhTEOS sol-gel film. For further experiments, we chose a film obtained by adding 1% PhTEOS to a mixture of precursors—HRP-PhTEOS1.

Three types of films were prepared for the following studies using this sol-gel matrix: HRP-PhTEOS1 with immobilized HRP, MT-PhTEOS1 with immobilized MT, BE-PhTEOS1 with immobilized BE. The relative activities were also calculated for MT and BE (Table 2) and they were similar to the HRP activities obtained in our study and other works [9,19]. No available data on the relative activity of immobilized tyrosinase or crude extracts were found in the literature.

HRP-PhTEOS1, MT-PhTEOS1, and BE-PhTEOS1 films were used to study the kinetics of enzymatic reactions.

#### 2.1.2. Sol-Gel Films Based on Silicon Polyethylene Glycol (SPG Films)

Another approach for creating biocomposite sol-gel films is the employment of silicon polyethylene glycol (SPG). SPG rapidly hydrolyzes and forms gels in aqueous media without the need for any catalyst, such as hydrochloric acid, to form silica hydrogels, which are transparent, and physically stable [21,22,23]. This approach was tested with many biological molecules, such as peroxidase, catalase, various oxidases, etc. [21]. We decided to synthesize glycerol-containing-precursors based sol-gel films and compare them to our TEOS-PhTEOS films.

Unlike alkoxide-based films, no ethanol is generated when using SPG, so SPG films are easier to prepare. In order to obtain SPG films, the precursor was mixed with a solution of the enzyme/extract in a pH 6.0 buffer solution in a ratio of 1:2. To form sol-gel films, 0.6 mL of the mixture was placed in cuvettes, distributed on one of its inner sides, and after 90 min, films with an approximate thickness of 1.5–2.5 mm were formed. Gelation occurred in the absence of a catalyst, and film formation took longer than in the case of TEOS-PhTEOS films.

Three types of films were prepared for the following studies using this sol-gel matrix: HRP-SPG with immobilized HRP, MT-SPG with immobilized MT, BE-SPG with immobilized BE. The relative activity of enzymes in these films is given in Table 2, and it is comparable to TEOS-PhTEOS based films. HRP-SPG, MT-SPG, and BE-SPG films were also used to study the kinetics of enzymatic reactions.

### 2.2. Study of the Kinetics of Enzymatic Reactions in the Presence of Sol-Gel Films Doped with HRP, MT, and BE

The effect of the sol-gel process on the enzyme activity was investigated by comparing the kinetic parameters (Michaelis constants) of the reactions catalyzed by native and immobilized enzyme. The initial rates of the HRP, MT, and BE catalyzed reactions were measured as the absorbance increase over time. The Michaelis constants were obtained through the fitting of the data to Michaelis–Menten kinetic analysis using Lineweaver–Burk plots. The Michaelis constants were calculated for hydrogen peroxide in the presence of constant TMB concentration (0.009%) in the case of HRP and for caffeic acid in the cases of MT and BE.

**Figure 2:**
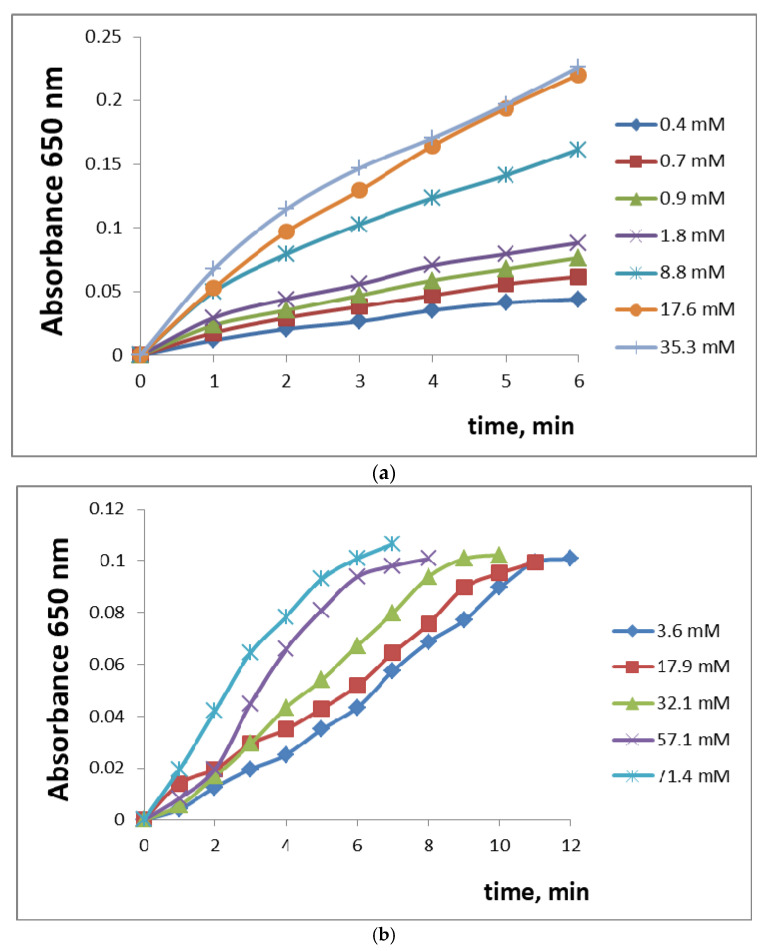
Kinetic curves of the TMB oxidation (absorbance at 650 nm over time) in the presence of different hydrogen peroxide concentrations (indicated in the legends) and HRP-PhTEOS1 (**a**) or HRP-SPG (**b**) sol-gel films.

The enzymes included in the sol-gel films obtained in this work retain their activity, and the kinetics of the reactions catalyzed can be fitted to the Michaelis–Menten equation. Figure 2 shows, for example, kinetic curves for different concentrations of hydrogen peroxide in the presence of HRP-PhTEOS1 and HRP-SPG films.

**Figure 3:**
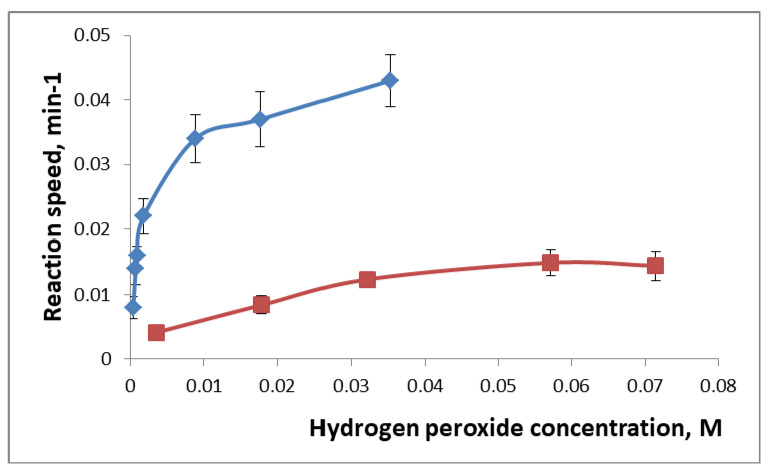
The dependence of the reaction speed (min^−1^) in the presence of HRP-PhTEOS1 film (blue) and HRP-PGS film (red) on the concentration of hydrogen peroxide (*n* = 3).

For the evaluation of the immobilized enzymes’ properties, we studied their interaction with substrates (hydrogen peroxide in the case of HRP and caffeic acid in the case of MT and BE) and calculated the kinetic parameters of the enzymatic reaction (Michaelis constants). Figure 3 shows the dependence of the reaction rate on the concentration of hydrogen peroxide in the presence of HRP-PhTEOS1 and HRP-SPG films. When Lineweaver–Burk coordinates are used, these dependencies become linear and allow the calculation of the Michaelis constants (Table 3). Both in our experiments and in the literature data [11,18,19] the Michaelis constant values (K_M_) of the immobilized enzymes were higher than those of the native enzymes, indicating the presence of partitioning and diffusional effects in the pores of the sol-gel matrix. Table 3 shows that, when using SPG-based films, an even greater increase in K_M_ values is observed, meaning that such films are better fit for the determination of high concentrations of substrates. This can be explained by the greater steric hindrance because of bulkier sol-gel precursor molecules. The obtained data indicate that for all the studied sol-gel films, the inclusion of HRP, MT, and BE does not hinder their enzymatic activity and allows their use for enzymatic reactions. TEOS-PhTEOS-based films seem to be more promising for the development of methods for determining low contents of analytes-substrates.

We have studied crude banana extract in the present immobilization study, because earlier we have established that crude plant extracts have higher interference thresholds than purified enzymes [24]. There are data indicating that crude extracts are more robust and endure sol-gel immobilization better: in some cases, the extract can withstand the immobilization procedure that inhibits the corresponding purified enzyme activity [11]. In the present study, we observed that for crude banana extract the Michaelis constant remained almost the same after the immobilization in PhTEOS1 film (2.4 mM in solution vs. 2.8 mM in film). A similar effect was described earlier for desert truffle tyrosinase extract [11]: the Michaelis constant even slightly decreased upon immobilization (0.5 mM in solution vs.0.2 mM in film). This can be possibly explained by the presence of other plant cell fragments in the crude extracts which create a better environment for the enzymes inside the sol-gel matrices.

Based on the enzyme kinetics study of the immobilized enzymes we have chosen HRP-PhTEOS1, MT-PhTEOS1, and BE-PhTEOS1 films for the analytical application.

### 2.3. Analytical Application HRP-PhTEOS1, MT-PhTEOS1, and BE-PhTEOS1 Films

We studied the possibility of the analytical use of the proposed sol-gel films doped with enzymes and banana extract. Hydrogen peroxide was used as analyte for HRP-PhTEOS1 film in the presence of TMB, and caffeic acid was used for MT-PhTEOS1 and BE-PhTEOS1 films. Immobilized enzymes catalyze the corresponding chromogenic reactions: hydrogen peroxide reduction with TMB oxidation and caffeic acid oxidation by air oxygen. The reaction rates were used as an analytical signal; the dependence of the absorbance on time was studied for different concentrations of analytes. Analytical ranges—analyte concentration ranges with a linear dependence of the reaction rate on analyte concentrations—are given in Table 4. Comparison of detection limits (LOD) for the sol-gel film encapsulated enzymes and banana extract, with LODs for non-immobilized enzymes and extract, demonstrate only 2–3 fold loss of sensitivity (Table 4). Such effect is likely attributed to steric hindrances arising during immobilization. The simplicity of determinations using sol-gel films doped with enzymes and banana extract should be noted: it is simply needed to place 3.0 mL of a sample in a cuvette containing a sol-gel film (in the case of determining hydrogen peroxide, 0.4 mL of 0.08% TMB solution should also be added), and monitor the change in absorbance at 650 nm for the determination of hydrogen peroxide and 400 nm for the determination of caffeic acid. Such measurements can be carried out using various portable photometers, which opens the prospect of mass analyses for the determination of biochemically active analytes in the field.

In this paper, to demonstrate the analytical capabilities of sol-gel films, we present the results of the total polyphenol content determination in coffee (in caffeic acid equivalents) using an immobilized banana extract (BE-PhTEOS1 film). Total polyphenol content determination is often used in food quality control [24].

The recovery study of the caffeic acid determination using BE-PhTEOS1 film shows that the RSD values are comparable to those for banana extract in solution and equal 7–10% (*n* = 3).

The results of TPC determination in coffee compared with the results of independent methods are given in Table 5.

The good agreement between the different procedures indicates the good accuracy of the TPC determination with BE-PhTEOS1 film. No significant difference was found between the four values using Student test (*p* > 0.28 for all the pairs).

The stability and lifetime of the immobilized BE was investigated by measuring the sol-gel film activity using 2.0 mM caffeic acid solution. 95% activity of immobilized BE was retained after 2 months storage at +4 °C (BE solution lost its activity after 4 days). 90% activity of immobilized BE was retained after 2 weeks storage at +25 °C.

These novel enzyme-doped silica matrixes provide promising platforms for development of various on-site analytical procedures. In the present work we proposed a procedure using crude plant extract immobilized in TEOS-PhTEOS sol-gel film (BE-PhTEOS1 film) for spectrophotometric determination of total polyphenol content using a standard curve of caffeic acid. The detection limit for caffeic acid equals 0.7 mM, while LOD values of other enzymatic methods of TPC determination lie in the 0.01–0.5 mM range [24]. However, the sol-gel films are ready-to-use and offer the possibility of storage at a room temperature. Using crude extract as the enzyme source in these sol-gel materials allows low-cost analysis which makes the process suitable for wide screening tests.

## 3. Conclusions

We have chosen the conditions for the synthesis of sol-gel films doped with HRP, MT, and BE using TEOS-PhTEOS mixture or SPG as precursors, on the inner side surface of polystyrene cuvettes. When using TEOS and PhTEOS precursors, the film preparation consists of two stages: the preparation of the sol under the influence of ultrasound for 90 min, which leads to the evaporation of a significant part of the formed alcohol, and the subsequent mixing of the sol with an enzyme solution. In the case of SPG films, the enzyme solution is mixed directly with the precursor. Basing on the study of the activity of the immobilized enzymes and the immobilized extract, we have concluded that for both types of films, the enzymatic activity is preserved, and the kinetics of the catalyzed reactions can be described by the Michaelis–Menten equation. The relative activity of the immobilized enzymes is comparable for both types of films and is about 10% of the activity of the non-immobilized enzyme. Thus, the preservation of enzyme activity in the proposed procedures is comparable to those described in the literature, which can also sometimes be significantly more complicated in execution.

When HRP and MT are included in alkoxide-based films, the Michaelis constants increase 3–4 fold, and in SPG-based films—10–20 fold. Compared to these purified enzymes, the crude banana extract demonstrates that it can better withstand the effect of immobilization: for BE the Michaelis constant almost does not change in the alkoxide-based films, and it increases only 3 fold in SPG-based films.

The analytical capabilities of sol-gel films doped with enzymes and banana extract are demonstrated: the analytical range for the hydrogen peroxide determination is 0.2–3.5 mM using HRP-PhTEOS1 film in the presence of TMB, and the analytical ranges for caffeic acid determination are 0.5–10.0 mM and 2.0–10.0 mM using MT-PhTEOS1 and BE-PhTEOS1 films, respectively. The sensitivity of the determination is decreased only 2–3 fold compared to non-immobilized enzymes, while the use of disposable cuvettes with a sol-gel film on the inner side surface greatly simplifies the determination procedure and makes it possible to carry out the determination in field conditions.

BE-PhTEOS1 films have been used to determine the total polyphenol content of coffee in caffeic acid equivalents. The lifetime of BE-PhTEOS1 is 2 months at +4 °C storage and 2 weeks at +25 °C storage, which is a significant improvement of the shelf life compared to the non-immobilized enzymes and extracts.

## 4. Materials and Methods

### 4.1. Materials

Tetraethoxysilane (TEOS), phenyltriethoxysilane (PhTEOS), 3-aminopropyltriethoxy-silane (AmTEOS), and hydrogen peroxide were purchased from Sigma-Aldrich (St. Louis, MO, USA). Silicon polyethylene glycol (SPG) was synthesized under the supervision of Dr. T.G. Khonina according to the method described earlier [22].

Caffeic acid, cetylpyridinium chloride, and 3,3′,5,5′-tetramethylbenzidine (TMB) were purchased from Acros Organics (Carlsbad, CA, USA). Phosphate buffer (pH 6.0) was prepared using sodium monophosphate and potassium diphosphate.

Horseradish peroxidase (HRP) (250 U/mg) was purchased from Biozyme laboratories (UK). Mushroom tyrosinase (MT) from *A. niger* (3900 U/mg) was purchased from Sigma (St. Louis, MO, USA).

Banana extract (BE) was prepared similarly to [24]: 100.0 g of homogenized banana pulp tissue was stirred in 200.0 mL of phosphate buffer (pH 6.0) at 0 °C for 30 min, and then filtered twice through a paper filter. The protein content of the banana extract was determined by the Biuret method. Total protein content equaled 3.8 mg/mL for banana pulp crude extract. The activity of the crude banana extract used in this work has been determined by comparing the reaction speed of catechol oxidation in the presence of the crude extract and the commercial mushroom tyrosinase. Crude banana extract activity was found to be 292 ± 6 U/mL (*n* = 3, *P* = 0.95).

Polystyrene cuvettes (10 × 10 × 45 mm) with caps were purchased from Sarstedt (Numbrecht, Germany).

### 4.2. Synthesis of Sol-Gel Films Doped with Mushroom Tyrosinase, Horseradish Peroxidase and Banana Extract

#### 4.2.1. Sol-Gel Films Based on TEOS and Its Mixtures with PhTEOS and AmTEOS (TEOS film)

In vials, a certain volume of AmTEOS or PhTEOS was added to a certain volume of TEOS. An aqueous solution of cetylpyridinium chloride and hydrochloric acid as a catalyst was added to the resulting mixture. The silane mixture: water ratio was 3:1. The mixture was stirred under the influence of ultrasound with the sound energy density of 0.28–0.34 W/mL for 90 min. In a plastic cuvette, 0.3 mL of the sol was mixed with 0.3 mL of the enzyme/crude extract solution in buffer. The cuvettes were capped, shaken, and then placed on their side. After 2–10 min, a transparent sol–gel film formed on the inner wall surface of the cuvette. The cuvettes with films were stored at +4 °C.

#### 4.2.2. Synthesis of HRP-PhTEOS1, MT-PhTEOS1, BE-PhTEOS1 Films

1.5 mL of aqueous solution containing 0.5 mMcetylpyridinium chloride and 1.6 mM hydrochloric acid was added to 4.5 mL of TEOS and 0.05 mL of PhTEOS. The mixture was stirred under the influence of ultrasound with a sound energy density of 0.3 W/mL for 90 min. In a plastic cuvette, 0.3 mL of the sol was mixed with 0.3 mL of HRP solution in buffer (pH 6.0) to obtain HRP-PhTEOS1 film, with 0.3 mL MT solution in buffer (pH 6) to obtain MT-PhTEOS1 film, 0.3 mL of BE to obtain BE-PhTEOS1 film. The cuvettes were capped, shaken, and then placed on their side. After 2–10 min, a transparent sol–gel film formed on the inner wall surface of the cuvette. The cuvettes with films were stored at +4 °C.

#### 4.2.3. Sol-Gel Films Based on SPG

In a cuvette 0.2 mL of SPG was mixed with 0.4 mL of HRP solution in buffer (pH 6.0) to obtain an HRP-SPG film, with 0.4 mL of MT solution in buffer (pH 6.0) to obtain a MT-SPG film, with 0.4 mL of BE to obtain a BE-SPG film. The cuvettes were capped, shaken, and then placed on their side. After 60–90 min, a transparent sol–gel film formed on the inner wall surface of the cuvette. The cuvettes with films were stored at +4 °C.

### 4.3. Study of the Immobilized HRP, MT, and BE Activity and Properties in Sol-Gel Films

#### 4.3.1. Relative Activity of Immobilized Enzymes

To study the activity of HRP immobilized in sol-gel films, 3.0 mL of a hydrogen peroxide solution of various concentrations and 0.4 mL of a 0.08% TMB solution were added to the cuvette with film. Absorbance was measured at 650 nm for 10 min every 10 s. Enzyme activity was determined as the initial reaction rate. The relative activity was determined from the dependence of the HRP activity in solution on the HRP amount. This dependence was obtained by the following procedure: 3.0 mL of 8.0 mM hydrogen peroxide solution was mixed with 0.4 mL of 0.08% TMB solution and 0.4 mL of HRP solution with different amounts of enzyme, and absorbance was measured at 650 nm.

To study activity of MT and BE immobilized in sol-gel films, 3.0 mL of caffeic acid solution of various concentrations was added to the cuvette with film and the absorbance was measured at 400 nm for 10–15 min every 10 s. Enzyme activity was determined as the initial reaction rate. The relative activity was determined from the dependence of the MT/BE activity in the solution on the MT/BE amount. This dependence was obtained by the following procedure: 1.0 mL of MT/BE solution with different amounts of enzyme was added to 2.0 mL of 5.0 mM caffeic acid solution, and the absorbance was measured at 400 nm.

#### 4.3.2. HRP Retention on PhTEOS0, PhTEOS1, PhTEOS2 Films

To study the retention of peroxidase in films of various compositions—TEOS (PhTEOS0), TEOS + 1%PhTEOS (PhTEOS1), TEOS + 2%PhTEOS (PhTEOS2)—2.0 mL of a buffer solution (pH 6.0) was added to the cuvettes with films and left for 30 min. After that, the buffer solution was decanted and its enzyme activity was determined according to the described above procedure.

#### 4.3.3. Sol-Gel Films Stability Studies

The films doped with enzymes and banana extract were stored in closed cuvettes at +25 °C and at +4 °C; their stability was checked by measuring the activity according to the described above procedure.

#### 4.3.4. Study of the Kinetics of Enzymatic Reactions in the Presence of Immobilized HRP, MT, and BE

To study the activity of HRP immobilized in sol-gel films, 3.0 mL of a hydrogen peroxide solution of various concentrations and 0.4 mL of a 0.08% TMB solution were added to the cuvette. Absorbance was measured at 650 nm for 10 min every 10 s. To calculate the Michaelis constant, the dependence of the reaction rate (min^−1^) on the concentration of hydrogen peroxide was plotted in Lineweaver–Burk coordinates.

To study the activity of MT and BE immobilized in sol-gel films, 3.0 mL of caffeic acid solution of various concentrations was added to the cuvette and the absorbance was measured at 400 nm for 10–15 min every 10 s. To calculate the Michaelis constant, the dependence of the reaction rate (min^−1^) on the concentration of caffeic acid was plotted in Lineweaver–Burk coordinates.

### 4.4. Calibration Curves Using HRP-PhTEOS1, MT-PhTEOS1, BE-PhTEOS1 Films

To obtain a calibration curve for hydrogen peroxide, 3.0 mL of a hydrogen peroxide solution of various concentrations and 0.4 mL of a 0.08% TMB solution were added to a cuvette with HRP-PhTEOS1 film. The difference in absorbance at 650 nm, measured after 1 and 2 min from the reaction start, was used as analytical signal.

To obtain a calibration curve for caffeic acid, 3.0 mL of caffeic acid solution of various concentrations was added to a cuvette with MT-PhTEOS1 or BE-PhTEOS1 films. The reaction rate, i.e., the rate of increase in absorbance at 400 nm, was used as analytical signal.

The limit of detection (LOD) was calculated as 3 standard deviation of the blank absorbance (*n* = 3) divided by the slope value. The limit of quantitation (LOQ) was calculated as 3·LOD.

### 4.5. Total Polyphenol Content Determination

1.0 g of coffee sample was mixed with 100.0 mL of boiling water, and filtered after 15 min. After cooling to the room temperature, 3.0 mL of the sample solution was added to the cuvette with the BE-PhTEOS1 film, and the reaction rate was used as the analytical signal. The total polyphenol content (TPC) in caffeic acid equivalents was determined in the treated sample using the standard addition method and using the calibration curve for caffeic acid in the range of 2.0–10.0 mM.

The procedure for TPC determination with Folin reagent was carried out similarly to [24].

### 4.6. Instrumentation

Sols were prepared under the ultrasound radiation using the ultrasound equipment UZH-02 (SonoTech, Russia). The sound energy density (W/mL) was defined as the ratio of the power absorbed in the reactor to the volume of liquid in the reactor. To determine the power, the time was measured until a certain mass of water was heated to a certain temperature.

Spectra of colored products of enzymatic oxidation of phenolic compounds were recorded with SPECTROstar Nano spectrophotometer (BMG Labtech, Ortenberg, Germany). Spectra were analyzed with MARS software (BMG Labtech, Ortenberg, Germany) and statistical analysis was carried out using MS Excel.

## Figures and Tables

**Table 1:** Retention of HRP in various sol-gel films after washing.

Precursor(s), Biocomposite Sol-Gel Material Preparation Procedure	Retention, %	Reference
TMOS, enzyme is added to the hydrolyzing mixture	76–90	[21]
PGS, enzyme is added to the hydrolyzing mixture	83–95	
Sodium silicate, ion-exchange elimination of sodium at the sol formation stage, enzyme is added to the sol	100	[19]
TEOS, enzyme is added to the sol	41	Present work
TEOS and 1% PhTEOS, enzyme is added to the sol	81	
TEOS and 2% PhTEOS, enzyme is added to the sol	79	

**Table 2:** Relative activity of the immobilized enzymes (% of the activity of the same amount of native enzyme) for the different sol-gel films.

Enzyme	Precursor(s), Biocomposite Sol-Gel Material Preparation Procedure	Enzyme Relative Activity, % (*n* = 3, *P* = 0.95)	Reference
HRP	Sodium silicate, ion-exchange elimination of sodium at the sol formation stage, enzyme is added to the sol	7.2	[19]
	TEOS, enzyme sorption on the nascent sol-gel film on the glass slide	11.7 ± 0.5	[9]
	TEOS and PhTEOS, enzyme is added to the sol, 1. 0% PhTEOS 2. 1% PhTEOS 3. 2% PhTEOS	6.6 ± 0.5 7.4 ± 0.6 6.9 ± 0.5	Present work
	SPG, enzyme is added to the hydrolyzing mixture	3.4 ± 0.4	
MT	TEOS and PhTEOS, enzyme is added to the sol, 1% PhTEOS	11.2 ± 1.0	
	SPG, enzyme is added to the hydrolyzing mixture	15.2 ± 2.2	
BE	TEOS and PhTEOS, enzyme is added to the sol,1% PhTEOS	10.8 ± 0.9	
	SPG, enzyme is added to the hydrolyzing mixture	8.5 ± 0.5	

HRP—horseradish peroxidase, MT—mushroom tyrosinase, BE—crude banana extract.

**Table 3:** The influence of immobilization in sol-gel film on the Michaelis constants (K_M_).

Enzyme Source	Film Precursor(s)	K_M_, mM		K_M_ Ratio	Reference
		Solution	Film		
Horseradish peroxidase	Na silicate	0.163	0.985	6	[19]
	TEOS	0.55	2.38	4.3	[18]
	TEOS+ 1% PhTEOS	0.4	1.4	3.5	Present work
	SPG	0.4	9.9	24.8	
Mushroom tyrosinase	TEOS + colloidal silica	0.9	no activity	-	[11]
	TEOS+ 1% PhTEOS	1.1	4.1	3.7	Present work
	SPG	1.1	10.1	9.2	
Desert truffle extract	TEOS + colloidal silica	0.5	0.2	0.4	[11]
Banana extract	TEOS+ 1% PhTEOS	2.4	2.8	1.2	Present work
	SPG	2.4	8.0	3.3	

**Table 4:** Analytical parameters of the procedures using HRP-PhTEOS1, MT-PhTEOS1, and BE-PhTEOS1 sol-gel films.

	Enzyme Source	Analytical Range, mM	LOD, mM (*n* = 3)
Hydrogen peroxide	HRP	0.05–0.5	0.02
	HRP-PhTEOS1	0.2–3.5	0.06
Caffeic acid	MT	0.25–10.0	0.08
	MT-PhTEOS1	0.5–10	0.15
	BE	0.5–10.0	0.2
	BE-PhTEOS1	2.0–10.0	0.6

**Table 5:** Results of TPC determination in coffee using BE-PhTEOS1 film and by independent methods (*n* = 3, *P* = 0.95).

Found, mg/g			
BE—PhTEOS 1		BE	Folin’s Reagent
Method of Standard Addition	Using the Calibration Curve		
114 ± 18	118 ± 36	110 ± 30	120 ± 10

## Data Availability

No data is available.
